# Demographics and treatment of patients with primary nephrotic syndrome in Japan using a national registry of clinical personal records

**DOI:** 10.1038/s41598-023-41909-5

**Published:** 2023-09-07

**Authors:** Naoki Nakagawa, Tomonori Kimura, Ryuichi Sakate, Takehiko Wada, Kengo Furuichi, Hirokazu Okada, Yoshitaka Isaka, Ichiei Narita

**Affiliations:** 1 Division of Cardiology, Nephrology, Pulmonology and Neurology, Department of Internal Medicine, Asahikawa Medical University, 2-1-1-1 Midorigaoka-higashi, Asahikawa, Japan; 2 Reverse Translational Research Project, Center for Rare Disease Research, National Institutes of Biomedical Innovation, Health and Nutrition (NIBIOHN), Ibaraki, Japan; 3 Laboratory of Rare Disease Resource Library, Center for Rare Disease Research, National Institutes of Biomedical Innovation, Health and Nutrition (NIBIOHN), Ibaraki, Japan; 4 Division of Nephrology, Endocrinology and Metabolism, Tokai University School of Medicine, Isehara, Japan; 5 Department of Nephrology, Toranomon Hospital, Tokyo, Japan; 6 Department of Nephrology, Kanazawa Medical University School of Medicine, Uchinada, Japan; 7 Department of Nephrology, Faculty of Medicine, Saitama Medical University, Saitama, Japan; 8 Department of Nephrology, Osaka University Graduate School of Medicine, Suita, Japan; 9 Division of Clinical Nephrology and Rheumatology, Kidney Research Center, Niigata University Graduate School of Medical and Dental Sciences, Niigata, Japan

**Keywords:** Focal segmental glomerulosclerosis, Membranous nephropathy, Minimal change disease

## Abstract

The nationwide clinical features of Japanese patients with primary nephrotic syndrome (NS), including minimal change disease (MCD), focal segmental glomerulosclerosis (FSGS), or membranous nephropathy (MN), have not yet been reported. We collected the clinical personal records of patients with primary NS between 2015 and 2018 from the national registry organized by the Japanese Ministry of Health, Labour, and Welfare. Overall, the demographics, chronic kidney disease classification based on glomerular filtration rate and albuminuria, and treatment of 6036 patients were collected: 3394 (56.2%) with MCD, 677 (11.2%) with FSGS, 1455 (24.1%) with MN, and 510 (8.5%) with others. MN patients were older than MCD and FSGS patients (67 vs. 42 and 47 years, respectively). Steroid-dependent NS or frequently relapsing NS was found in 70.2%, 40.5%, and 24.6%, whereas steroid-resistant NS was found in 6.4%, 36.0%, and 37.9% of patients in the MCD, FSGS, and MN, respectively. The present oral prednisolone use (mean dose, mg/day) was 87.2% (21.2), 80.9% (20.0), and 77.5% (18.8) of patients in the MCD, FSGS, and MN, respectively. The national registry of clinical personal records of primary NS could provide an informative insight into the characteristics, clinical features, and treatment approaches for patients with primary NS in Japan.

## Introduction

Primary nephrotic syndrome (NS), a major course of nephrotic syndrome, is characterized by massive proteinuria, edema, and hypoalbuminuria^1,2^. Based on biopsy results, NS is classified into minimal change disease (MCD), focal segmental glomerulosclerosis (FSGS), and membranous nephropathy (MN)^3,4^ with the global incidence of 0.2–0.8, 0.2–1.1 and 0.3–1.4 per 100,000 person-years, respectively^5^. The Japan Nephrotic Syndrome Cohort Study (JNSCS), which enrolled 374 Japanese patients with primary NS, reported that the incidence of MCD, FSGS, MN, and others was 41.4%, 10.2%, 39.6% and 8.8%, respectively^6^, suggesting that their prevalence in Japan is similar to that worldwide. Furthermore, several studies from the Japan Renal Biopsy Registry (J-RBR), a nationwide, web-based registry of renal biopsies, have also described the clinical features of several primary NS in Japan^7–11^. These registries are useful for understanding the demographic, clinical characteristics, and treatment outcomes of several kidney diseases^6,12–14^. However, in Japan, most studies on primary NS are either regional or conducted on a small scale. Additionally, no cohort study with more than 1000 patients has reported the distribution of patients with primary NS. Therefore, large-scale demographics, clinical characteristics, and treatment data of Japanese patients with primary NS are needed for better healthcare services, to improve treatment, and to provide a reference for comparisons with future studies and medical administration.

Recent attention in medical and healthcare research has focused on large-scale data from various sources, including administrative or claims data, electronic health records, and data from self-monitoring devices^15–17^. Clinical personal records is a nationwide administrative database of public expenditure on intractable disease maintained by the Japanese Ministry of Health, Labour, and Welfare to register and certify intractable diseases^18^. This database started to collect data on primary NS data in 2015, which helped investigate the clinical features of routine practice for primary NS. The aim of this study was to describe the demographics, subtypes of glomerulopaties, and treatment of primary NS in Japan using a nationwide registry of clinical personal records.

## Results

**Figure 1:**
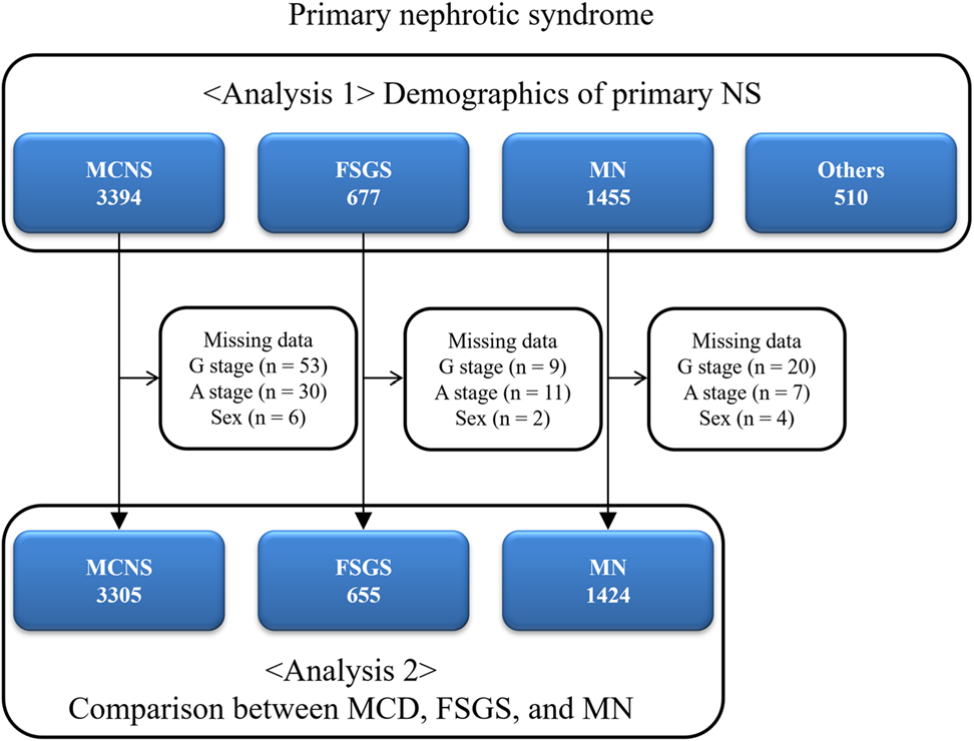
Flow diagram for patient selection. *MCD* minimal change disease, *FSGS* focal segmental glomerulosclerosis, *MN* membranous nephropathy.

Data were collected from 6036 patients with primary NS from 2015 to 2018 (Fig. 1). After rexcluding patients with unknown age of onset of ailment (n = 298), the pathologies included were as follows: MCD (n = 3394 [56.2%]), FSGS (n = 677 [11.2%]), MN (n = 1455 [24.1%]), membranoproliferative glomerulonephritis (n = 139 [2.3%]), crescentic glomerulonephritis (n = 19 [0.3%]), endocapillary proliferative glomerulonephritis (n = 13 [0.2%]), unknown pathologies (n = 232 [3.8%]), and unclassifiable diagnoses (n = 107 [1.8%]).

### Analysis 1: distribution and frequency of glomerulopathies in the national registry of clinical personal records of primary NS based on age of onset

**Figure 2:**
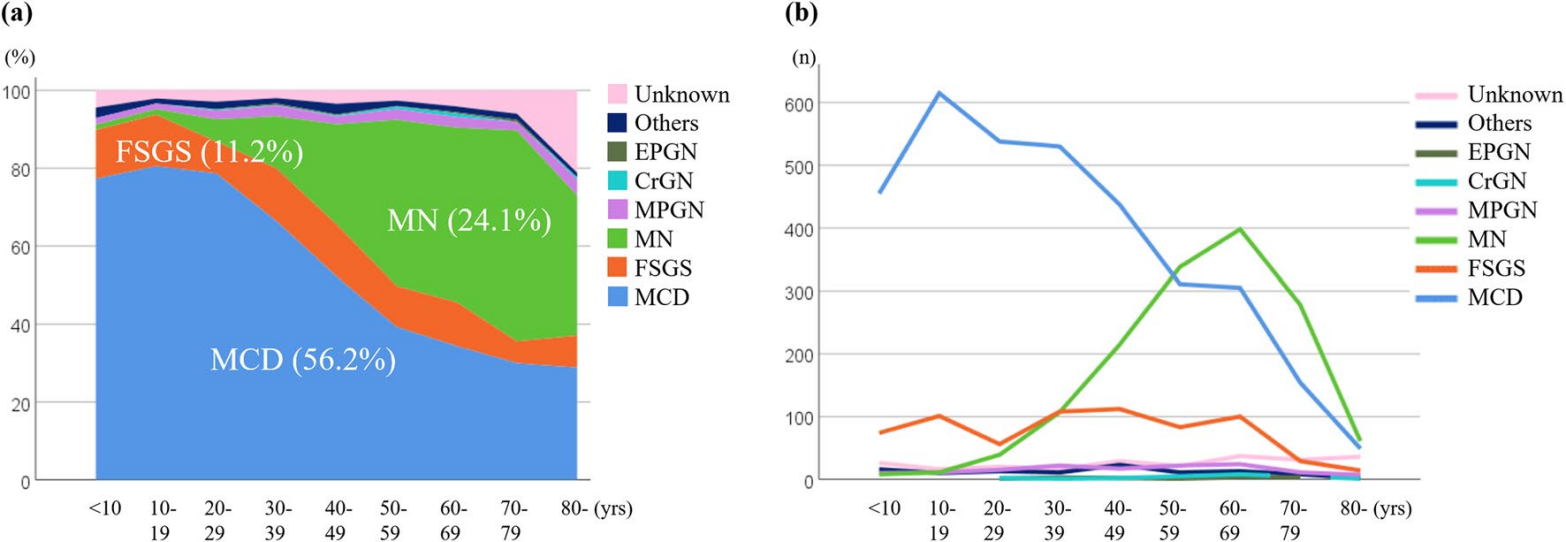
Distribution (**a**) and frequency (**b**) of glomerulopathies in the national registry of clinical personal records of primary nephrotic syndrome by age (in years) of onset. Minimal-change disease (MCD) was most common in younger ages, membranous nephropathy (MN) was primarily present in adults, and focal segmental glomerulosclerosis (FSGS) and other glomerulopathies were present in all ages. *MPGN* membranoproliferative glomerulonephritis, *CrGN* crescentic glomerulonephritis, *EPGN* endocapillary proliferative glomerulonephritis.

Regarding the age distribution, MCD is most common in child, adolescent, and young adults, whereas MN is most common in older adults. FSGS and other diagnoses were present in all ages (Fig. 2a and b).

### Analysis 2–1: comparison between MCD, FSGS, and MN

**Figure 3:**
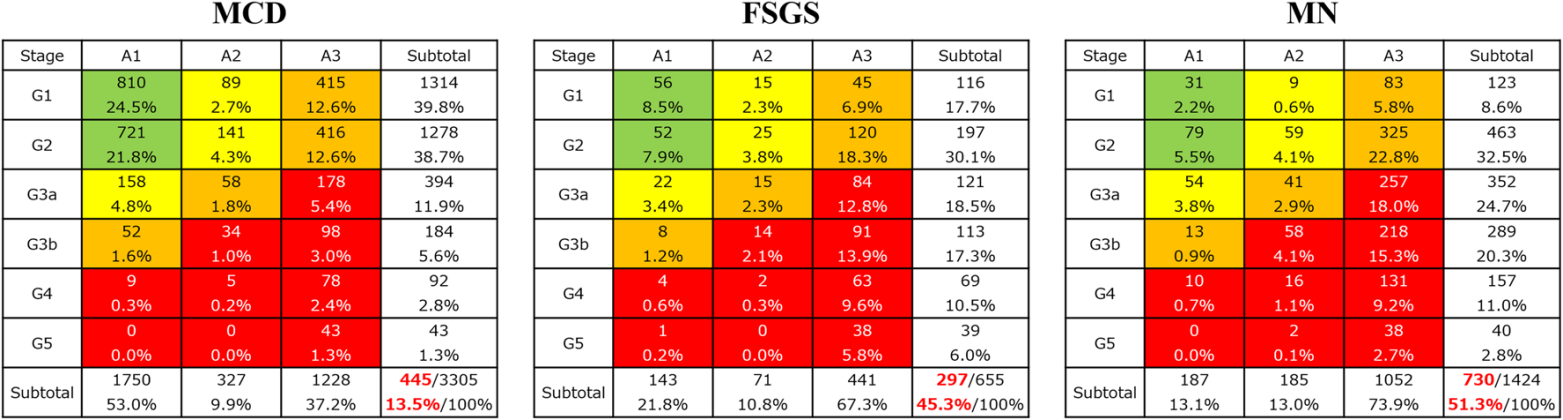
Risk classification of chronic kidney disease in patients with minimal-change disease (MCD), focal segmental glomerulosclerosis (FSGS), and membranous nephropathy (MN).

After removing patients with missing data, 3305 patients with MCD, 655 with FSGS, and 1424 with MN were included in Analysis 2 (Fig. 1, Table 1). The median (IQR) age of onset was lower for MCD as compared with FSGS and MN (31 [16–49] vs. 39 [19–55] and 60 [49–69] years, respectively; P < 0.001; Table 1). The median (IQR) age at renal biopsy and the median (IQR) age of enrollment were also lower for MCD than for FSGS and MN (34 [20–50] vs. 41 [24–58] and 61 [51–70] years, respectively; P < 0.001; 42 [29–56] vs. 47 [33–62] and 67 [58–74] years, respectively; P < 0.001; Table 1). However, the number of missing values was 353 (10.7%), 23 (3.5%), and 40 (2.8%), respectively. Male predominance was observed for MCD, FSGS, and MN. In accordance with the risk classification of chronic kidney disease, very high-risk (red zone) patients accounted for 13.5% of MCD cases, 45.3% of FSGS cases, and 51.3% of MN cases (Fig. 3).

**Table 1:** Comparison of demographics and treatment between patients with MCD, FSGS, and MN.

N	MCD	FSGS	MN	*P*-value
	3305	655	1424	
Age of onset (yrs)	31 (16, 49)	39 (19, 55)	60 (49, 69)	< 0.001^a,b,c^
Age at renal biopsy (yrs)*	34 (20, 50)	41 (24, 58)	61 (51, 70)	< 0.001^a,b,c^
Age of enrollment (yrs)	42 (29, 56)	47 (33, 62)	67 (58, 74)	< 0.001^a,b,c^
Male, n (%)	1899 (57.5%)	378 (57.5%)	911 (64.0%)	< 0.001^b,c^
Present treatment				
Oral PSL	2881 (87.2%)	530 (80.9%)	1104 (77.5%)	< 0.001^a,b^
IV mPSL	256 (7.7%)	73 (11.1%)	42 (2.9%)	< 0.001^a,b,c^
Cyclosporine	1559 (47.2%)	337 (51.5%)	621 (43.6%)	0.003^c^
Tacrolimus	51 (1.5%)	26 (4.0%)	15 (1.1%)	< 0.001^a,c^
Cyclophosphamide	16 (0.5%)	3 (0.5%)	20 (1.4%)	0.002^b^
Mizoribine	403 (12.2%)	81 (12.4%)	238 (16.7%)	< 0.001^b,c^
MMF	86 (2.6%)	29 (4.4%)	1 (0.1%)	< 0.001^a,b,c^
Rituximab	199 (6.0%)	38 (5.8%)	10 (0.7%)	< 0.001^b,c^
PSL + cyclosporine	1395 (42.2%)	299 (45.6%)	525 (36.9%)	< 0.001^b,c^
Dosage of present treatment				
Oral PSL (mg/day)	21.2 ± 17.8	20.0 ± 16.9	18.8 ± 15.4	0.001^b^
IV mPSL (mg/day)	568.7 ± 316.5	557.5 ± 219.7	558.7 ± 241.9	0.993
Cyclosporine (mg/day)	97.0 ± 40.8	95.8 ± 42.4	93.7 ± 46.0	0.149
Tacrolimus (mg/day)	2.4 ± 1.0	3.3 ± 2.1	2.5 ± 0.6	0.099
Cyclophosphamide (mg/day)	76.6 ± 33.5	83.3 ± 57.7	65.0 ± 22.1	0.349
Mizoribine (mg/day)	150.8 ± 66.2	148.3 ± 68.4	138.0 ± 42.7	0.144
MMF (mg/day)	1459.3 ± 521.4	1456.9 ± 586.4	750	0.359
Rituximab (mg/month)	594.3 ± 407.5	811.2 ± 612.9	511.1 ± 33.3	0.112
Steroid‑resistant NS	213 (6.4%)	236 (36.0%)	540 (37.9%)	< 0.001^a,b^
SDNS or FRNS	2320 (70.2%)	265 (40.5%)	351 (24.6%)	< 0.001^a,b,c^
Persistent proteinuria ≥ 0.5 g/gCr	583 (17.6%)	326 (49.8%)	828 (58.1%)	< 0.001^a,b,c^

Data are expressed as median (interquartile range), mean ± SD or number (percentage).

*MCD* minimal change disease, *FSGS* focal segmental glomerulosclerosis, *MN* membranous nephropathy, *NS* nephrotic syndrome, *PSL* prednisolone, *mPSL* methylprednisolone, *MMF* Mycophenolate mofetil, *SDNS* steroid-dependent nephrotic syndrome, *FRNS* frequently relapsing nephrotic syndrome.

^a^P < 0.05, MCD vs. FSGS, ^b^P < 0.05, MCD vs. MN, ^c^P < 0.05, FSGS vs. MN. Kruskal–Wallis tests with Bonferroni-corrected P-values.

*Number of missing values, n = 353 (10.7%) in MCD; n = 23 (3.5%) in FSGS; n = 40 (2.8%) in MN.

The use of oral prednisolone was significantly higher in MCD (87.2%) than in FSGS (80.9%) and MN (77.5%) (Table 1). The mean dose of oral prednisolone in MCD (21.2 ± 17.8 mg/day) was significantly higher than in MN (18.8 ± 15.4 mg/day). However, the use of intravenous methylprednisolone was significantly higher in FSGS (11.1%) than in MCD (7.7%) and MN (2.9%). Regarding the immunosuppressive drugs used to treat each pathological type of NS, cyclosporine was most commonly used to treat MCD, FSGS, and MN (47.2%, 51.5%, and 43.6%, respectively), followed by mizoribine (12.2%, 12.4% and 16.7%, respectively), rituximab (6.0%, 5.8% and 0.7%, respectively), and mycophenolate mofetil (2.6%, 4.4% and 0.1%, respectively). Prednisolone + cyclosporine combination therapy was used to treat MCD, FSGS, and MN (42.2%, 45.6% and 36.9%, respectively). The use of mizoribine was significantly higher in MN than in MCD. In contrast, rituximab and prednisolone + cyclosporine combination therapy was significantly lower in MN than in MCD and FSGS (Table 1).

As for steroid responses, the steroid-resistant NS was significantly higher in MN and FSGS than in MCD (37.9%, 36.0% and 6.4%, respectively). In the steroid-resistant NS, 213 patients had MCD, 236 had FSGS, and 540 had MN (Supplementary Table S1). Although the present use of oral prednisolone and cyclosporine was not different among the three glomerulopathies, the present use of mizoribine was significantly higher in MN (22.8%) than in MCD (10.8%) (Supplementary Table S1).

In contrast, steroid-dependent nephrotic syndrome (SDNS) or frequently relapsing nephrotic syndrome (FRNS) was significantly higher in MCD than in FSGS and MN (70.2%, 40.5%, and 24.6%, respectively) (Table 1). In the SDNS or FRNS, a total of 2320 patients had MCD, 265 had FSGS, and 351 had MN (Supplementary Table S2). Although the present use of oral prednisolone and mizoribine was not different among the three glomerulopathies, the highest use of intravenous methylprednisolone, cyclosporine, tacrolimus, mycophenolate mofetil, and rituximab was observed in patients with steroid-dependent or frequently relapsing FSGS (Supplementary Table S2).

### Analysis 2–2: comparison among four age groups in MCD, FSGS, and MN

Patients were divided into five age groups: child and adolescent (≤ 19 years), young adult (20–39 years), middle adult (40–64 years), elderly (65–74 years), and very elderly (≥ 75 years). The distribution and risk classification of chronic kidney disease associated with MCD, FSGS, and MN are shown in Supplementary Fig. S1. MCD was dominant in the child and adolescent, young and middle adult groups, whereas MN was in the elderly and very elderly groups. According to the risk classification of chronic kidney disease, the frequency of very high-risk (red zone) patients with FSGS was the highest compared with that of MCD and MN in all five age groups.

In patients with MCD, the use of oral prednisolone and intravenous methylprednisolone was significantly lower in the child and adolescent groups as compared with the other age groups (Table 2). In contrast, the mean dosage of oral prednisolone was significantly higher in the elderly and very elderly groups (24.0 ± 18. 2 and 24.7 ± 17.4 mg/day, respectively) than in other age groups (Table 2). The use and the mean dosage of cyclosporine were significantly higher in child and adolescent MCD patients (51.9% and 106.3 ± 45.0 mg/day) than in other age groups. Moreover, mizoribine, rituximab, and mycophenolate mofetil were used to a significantly higher extent in child and adolescent MCD patients than in other age groups. As for steroid responses, steroid-resistant MCD was significantly lower in child and adolescent and young adult MCD patients than in other age groups, whereas SDNS or FRNS was significantly higher in child and adolescent and young adult MCD patients than in other age groups (Table 2).

**Table 2:** Demographic data of patients with MCD according to age of onset.

N	≤ 19 y/o	20–39 y/o	40–64 y/o	65–74 y/o	≥ 75 y/o	*P*-value
	1038	1042	895	212	118	
Age of onset (yrs)	11 (5, 16)	29 (24, 34)	50 (44, 57)	68 (67, 71)	79 (76, 82)	< 0.001^a,b,c,d,e,f,g,h,i^
Age at renal biopsy (yrs)*	17 (14, 19)	31 (25, 36)	51 (45, 58)	69 (67, 72)	79 (77, 83)	< 0.001^a,b,c,d,e,f,g,h,i^
Age of enrollment (yrs)	24 (20, 32)	38 (32, 44)	56 (49, 63)	71 (68, 74)	81 (78, 84)	< 0.001^a,b,c,d,e,f,g,h,i^
Male, n (%)	703 (67.7%)	551 (52.9%)	447 (49.9%)	131 (61.8%)	67 (56.8%)	< 0.001^a,b,h^
Present treatment						
Oral PSL	862 (83.0%)	927 (89.0%)	796 (88.9%)	187 (88.2%)	109 (92.4%)	< 0.001^a,b,d^
IV mPSL	57 (5.5%)	81 (7.8%)	79 (8.8%)	26 (12.3%)	13 (11.0%)	0.002^c^
Cyclosporine	539 (51.9%)	527 (50.6%)	376 (42.0%)	76 (35.8%)	41 (34.7%)	< 0.001^b,c,d,e,f,g^
Mizoribine	178 (17.1%)	106 (10.2%)	98 (10.9%)	15 (7.1%)	6 (5.1%)	< 0.001^a,b,c,d^
MMF	79 (7.6%)	7 (0.7%)	0 (0.0%)	0 (0.0%)	0 (0.0%)	< 0.001^a,b,c,d^
Rituximab	120 (11.6%)	48 (4.6%)	29 (3.2%)	2 (0.9%)	0 (0.0%)	< 0.001^a,b,c,d^
PSL + cyclosporine	471 (45.4%)	477 (45.8%)	338 (37.8%)	69 (32.5%)	40 (33.9%)	< 0.001^e,f^
Dosage of treatment						
Oral PSL (mg/day)	21.5 ± 19.1	19.2 ± 16.1	22.2 ± 17.9	24.0 ± 18.2	24.7 ± 17.4	0.002^e,f,g^
IV mPSL (mg/day)	545.5 ± 429.4	603.3 ± 294.1	564.4 ± 266.3	526.7 ± 302.1	552.1 ± 222.7	0.282
Cyclosporine (mg/day)	106.3 ± 45.0	95.4 ± 39.9	89.8 ± 34.9	84.4 ± 34.2	85.9 ± 30.5	< 0.001^a,b,c^
Mizoribine (mg/day)	164.3 ± 84.9	145.3 ± 50.9	135.7 ± 35.1	143.3 ± 17.6	108.3 ± 37.6	0.031
MMF (mg/day)	1516.0 ± 472.2	906.3 ± 680.6	–	–	–	0.014
Rituximab (mg/month)	585.4 ± 363.6	606.7 ± 456.6	630.1 ± 495.3	300.0 ± 282.8	–	0.374
Steroid‑resistant NS	25 (2.4%)	40 (3.8%)	82 (9.2%)	39 (18.4%)	27 (22.9%)	< 0.001^b,c,d,e,f,g,h^
SDNS or FRNS	915 (88.2%)	803 (77.2%)	500 (55.9%)	69 (32.5%)	33 (28.0%)	< 0.001^a,b,c,d,e,f,g,h^
Persistent UP ≥ 0.5 g/gCr	83 (8.0%)	154 (14.8%)	240 (26.8%)	67 (31.6%)	39 (33.1%)	< 0.001^a,b,c,e,f,g^

Data are expressed as median (interquartile range), mean ± SD or number (percentage).

*MCD* minimal change disease, *PSL* prednisolone, *mPSL* methylprednisolone, *MMF* Mycophenolate mofetil, *NS* nephrotic syndrome, *SDNS* steroid-dependent nephrotic syndrome, *FRNS* frequently relapsing nephrotic syndrome.

^a^P < 0.05, ≤ 19 y/o vs. 20–39 y/o, ^b^P < 0.05, ≤ 19 y/o vs. 40–64 y/o, ^c^P < 0.05, ≤ 19 y/o vs. 65–74 y/o, ^d^P < 0.05, ≤ 19 y/o vs. ≥ 75 y/o, ^e^P < 0.05, 20–39 y/o vs. 40–64 y/o, ^f^P < 0.05, 20–39 y/o vs. 65–74 y/o, ^g^P < 0.05, 20–39 y/o vs. ≥ 75 y/o, ^h^P < 0.05, 40–64 y/o vs. 65–74 y/o, ^i^P < 0.05, 40–64 y/o vs. ≥ 75 y/o. Kruskal–Wallis tests with Bonferroni-corrected *P*-values.

*Number of missing values, n = 155 (14.9%) in ≤ 19 y/o; n = 98 (9.4%) in 20–39 y/o; n = 60 (6.7%) in 40–64 y/o; n = 29 (13.7%) in 65–74 y/o; n = 11 (9.3%) in ≥ 75 y/o.

With regards to patients with FSGS, the use of mycophenolate mofetil and rituximab was significantly higher in the child and adolescent groups than in the middle adult and elderly groups, but the use of oral prednisolone and intravenous methylprednisolone was lower in the child and adolescent groups than in the other age groups (Table 3). Other immunosuppressive drugs showed no differences among five age groups (Table 3). Steroid-resistant FSGS was significantly higher in the very elderly group than in the child and adolescent, young adult, and middle adult group (75.0%, 28.0%, 35.3%, and 34.2%, respectively). In contrast, SDNS or FRDS was significantly higher in the child and adolescent as well as in the young and middle adult group as compared with the very elderly group (52.2%, 42.9%, 40.1%, and 0.0%, respectively) in patients with FSGS (Table 3).

**Table 3:** Demographic data of patients with FSGS according to age of onset.

N	≤ 19 y/o	20–39 y/o	40–64 y/o	65–74 y/o	≥ 75 y/o	*P*-value
	164	164	241	61	25	
Age of onset (yrs)	11 (5, 16)	32 (26, 36)	51 (44, 59)	68 (67, 70)	80 (77, 82)	< 0.001^a,b,c,d,e,f,g,h,i^
Age at renal biopsy (yrs)*	18 (14, 20)	34 (28, 38)	52 (46, 60)	69 (67, 71)	82 (78, 83)	< 0.001^a,b,c,d,e,f,g,h,i^
Age of enrollment (yrs)	24 (20, 33)	40 (33, 44)	58 (50, 63)	70 (67, 76)	82 (78, 84)	< 0.001^a,b,c,d,e,f,g,h,i^
Male, n (%)	100 (61.0%)	86 (52.4%)	148 (61.4%)	32 (52.5%)	12 (48.0%)	0.233
Present treatment						
Oral PSL	110 (70.1%)	131 (84.0%)	197 (83.1%)	50 (90.9%)	17 (85.0%)	0.002^a,b,c^
IV mPSL	8 (5.1%)	22 (14.1%)	21 (8.9%)	13 (23.6%)	3 (15.0%)	0.001^a,c,h^
Cyclosporine	82 (52.2%)	77 (49.4%)	128 (54.0%)	24 (43.6%)	11 (55.0%)	0.395
Mizoribine	27 (17.2%)	23 (14.7%)	21 (8.9%)	6 (10.9%)	2 (10.0%)	0.142
MMF	21 (13.4%)	2 (1.3%)	6 (2.5%)	0 (0.0%)	0 (0.0%)	< 0.001^a,b,c,d^
Rituximab	17 (10.8%)	12 (7.7%)	9 (3.8%)	0 (0.0%)	0 (0.0%)	0.007^c^
PSL + cyclosporine	70 (44.6%)	69 (44.2%)	111 (46.8%)	24 (43.6%)	10 (50.0%)	0.458
Dosage of treatment						
Oral PSL (mg/day)	18.0 ± 17.1	21.8 ± 16.1	17.2 ± 16.4	26.9 ± 16.8	28.1 ± 19.0	< 0.001^d,e,h,i^
IV mPSL (mg/day)	555.6 ± 300.5	609.6 ± 237.5	508.8 ± 210.2	541.7 ± 144.3	500.0 ± 0.0	0.554
Cyclosporine (mg/day)	98.9 ± 45.6	101.2 ± 39.9	91.5 ± 41.3	92.1 ± 38.3	92.3 ± 54.4	0.357
Mizoribine (mg/day)	145.4 ± 71.4	152.2 ± 53.3	156.7 ± 87.7	141.7 ± 20.4	75.0 ± 35.4	0.236
MMF (mg/day)	1631.0 ± 491.3	500.5 ± 706.4	1166.7 ± 516.4	–	–	0.025
Rituximab (mg/month)	824.4 ± 628.3	772.7 ± 606.8	833.3 ± 661.4	–	–	0.956
Steroid‑resistant NS	44 (28.0%)	55 (35.3%)	81 (34.2%)	30 (54.5%)	15 (75.0%)	< 0.001^c,d,g,h,i^
SDNS or FRNS	82 (52.2%)	67 (42.9%)	95 (40.1%)	16 (29.1%)	0 (0.0%)	< 0.001^c,d,g,i^
Persistent UP ≥ 0.5 g/gCr	60 (38.2%)	86 (55.1%)	110 (46.4%)	38 (69.1%)	17 (85.0%)	< 0.001^a,c,d,h,i^

Data are expressed as median (interquartile range), mean ± SD or number (percentage).

*FSGS* focal segmental glomerulosclerosis, *PSL* prednisolone, *mPSL* methylprednisolone, *MMF* Mycophenolate mofetil, *NS* nephrotic syndrome, *SDNS* steroid-dependent nephrotic syndrome, *FRNS* frequently relapsing nephrotic syndrome.

^a^P < 0.05, ≤ 19 y/o vs. 20–39 y/o, ^b^P < 0.05, ≤ 19 y/o vs. 40–64 y/o, ^c^P < 0.05, ≤ 19 y/o vs. 65–74 y/o, ^d^P < 0.05, ≤ 19 y/o vs. ≥ 75 y/o, ^e^P < 0.05, 20–39 y/o vs. 40–64 y/o, ^f^P < 0.05, 20–39 y/o vs. 65–74 y/o, ^g^P < 0.05, 20–39 y/o vs. ≥ 75 y/o, ^h^P < 0.05, 40–64 y/o vs. 65–74 y/o, ^i^P < 0.05, 40–64 y/o vs. ≥ 75 y/o. Kruskal–Wallis tests with Bonferroni-corrected *P*-values.

*Number of missing values, n = 7 (4.3%) in ≤ 19 y/o; n = 7 (4.3%) in 20–39 y/o; n = 7 (2.9%) in 40–64 y/o; n = 2 (3.3%) in 65–74 y/o; n = 0 (0.0%) in ≥ 75 y/o.

In patients with MN, the use of prednisolone and other immunosuppressive drugs did not differ much among the five age groups, although the mean dosages of oral prednisolone and cyclosporine were higher in the older age groups (P = 0.007 and 0.017 for trend, respectively) (Table 4). Steroid-resistant MN was also similar among the five age groups (Table 4).

**Table 4:** Demographic data of patients with MN according to age of onset.

N	≤ 19 y/o	20–39 y/o	40–64 y/o	65–74 y/o	≥ 75 y/o	*P*-value
	18	144	726	363	173	
Age of onset (yrs)	11 (5, 16)	29 (24, 34)	50 (44, 57)	68 (67, 71)	79 (76, 82)	< 0.001^b,c,d,e,f,g,h,i,j^
Age at renal biopsy (yrs)*	18 (11, 21)	35 (32, 39)	57 (50, 61)	70 (67, 72)	79 (76, 82)	< 0.001^b,c,d,e,f,g,h,i,j^
Age of enrollment (yrs)	24 (20, 32)	38 (32, 44)	56 (49, 63)	71 (68, 74)	81 (78, 84)	< 0.001^b,c,d,e,f,g,h,i,j^
Male, n (%)	9 (50.0%)	81 (56.3%)	487 (67.1%)	221 (60.9%)	113 (65.3%)	0.042
Present treatment						
Oral PSL	13 (76.5%)	120 (84.5%)	540 (75.6%)	286 (79.4%)	125 (73.5%)	0.129
IV mPSL	0 (0.0%)	5 (3.5%)	18 (2.5%)	11 (3.1%)	7 (4.1%)	0.712
Cyclosporine	8 (47.1%)	63 (44.4%)	324 (45.4%)	148 (41.1%)	69 (40.6%)	0.622
Mizoribine	2 (11.8%)	32 (22.5%)	118 (16.5%)	48 (13.3%)	31 (18.2%)	0.161
MMF	0 (0.0%)	0 (0.0%)	0 (0.0%)	1 (0.3%)	0 (0.0%)	0.571
Rituximab	0 (0.0%)	0 (0.0%)	4 (0.6%)	5 (1.4%)	1 (0.6%)	0.435
PSL + cyclosporine	7 (41.2%)	58 (40.8%)	271 (38.0%)	125 (34.7%)	55 (32.4%)	0.487
Dosage of treatment						
Oral PSL (mg/day)	13.2 ± 15.9	17.9 ± 15.9	17.7 ± 15.2	20.3 ± 15.4	21.5 ± 15.2	0.007
IV mPSL (mg/day)	-	833.3 ± 288.7	588.5 ± 263.6	465.9 ± 113.1	514.3 ± 261.0	0.109
Cyclosporine (mg/day)	75.6 ± 38.5	106.0 ± 44.0	91.9 ± 37.0	94.6 ± 37.2	91.9 ± 86.9	0.017^ g^
Mizoribine (mg/day)	141.7 ± 14.4	142.2 ± 46.0	136.0 ± 35.7	129.3 ± 28.2	145.8 ± 62.7	0.214
MMF (mg/day)			–	750.0	–	–
Rituximab (mg/month)	–	–	500.0 ± 0.0	500.0 ± 0.0	600.0	0.134
Steroid‑resistant NS	6 (35.3%)	67 (47.2%)	255 (35.7%)	133 (36.9%)	69 (40.6%)	0.210
SDNS or FRNS	6 (35.3%)	46 (32.4%)	212 (29.7%)	61 (16.9%)	20 (11.8%)	< 0.001f.^,g,h,i^
Persistent UP ≥ 0.5 g/gCr	8 (47.1%)	87 (61.3%)	410 (57.4%)	210 (58.3%)	102 (60.0%)	0.844

Data are expressed as median (interquartile range), mean ± SD or number (percentage).

*MN* membranous nephropathy, *PSL* prednisolone, *mPSL* methylprednisolone, *MMF* Mycophenolate mofetil, *NS* nephrotic syndrome, *SDNS* steroid-dependent nephrotic syndrome, *FRNS* frequently relapsing nephrotic syndrome.

^a^P < 0.05, ≤ 19 y/o vs. 20–39 y/o, ^b^P < 0.05, ≤ 19 y/o vs. 40–64 y/o, ^c^P < 0.05, ≤ 19 y/o vs. 65–74 y/o, ^d^P < 0.05, ≤ 19 y/o vs. ≥ 75 y/o, ^e^P < 0.05, 20–39 y/o vs. 40–64 y/o, ^f^P < 0.05, 20–39 y/o vs. 65–74 y/o, ^g^P < 0.05, 20–39 y/o vs. ≥ 75 y/o, ^h^P < 0.05, 40–64 y/o vs. 65–74 y/o, ^i^P < 0.05, 40–64 y/o vs. ≥ 75 y/o, ^j^P < 0.05, 65–74 y/o vs. ≥ 75 y/o.Kruskal–Wallis tests with Bonferroni-corrected *P*-values.

*Number of missing values, n = 0 (0.0%) in ≤ 19 y/o; n = 4 (2.8%) in 20–39 y/o; n = 19 (2.6%) in 40–64 y/o; n = 10 (2.8%) in 65–74 y/o; n = 10 (2.8%) in ≥ 75 y/o.

### Quality indicators (QIs) related to the treatment for primary NS

As for QIs related to the treatment for primary NS, the use of prednisolone + cyclosporine combination therapy in steroid‑resistant MCD, FSGS, and MN in the present treatment was 42.7%, 47.4%, and 42.2%, respectively, but 51.6%, 67.8%, and 58.6%, respectively, in the previous treatment (Supplementary Tables S1 and S3). In addition, the use of prednisolone and cyclosporine combination therapy in steroid-dependent or frequently relapsing MCD accounted for 48.8% in the present treatment and 71.0% as per the previous treatment (Supplementary Tables S2 and S3).

## Discussion

This study is the first to describe clinical features and real-world management of patients, throughout Japan suffering from primary NS with MCD, FSGS, and MN, with the age of onset ranging from childhood to the very elderly,and including both the use and the mean dosage of immunosuppressants, according to a national database of more than 6000 patients.

Analysis 1 described the distribution and frequency of glomerulopathies in the national registry of clinical personal records of primary NS by age of onset. MCD, FSGS, and MN median ages were 31, 39, and 60 years, respectively. and younger than those of included in JNSCS (44, 59, and 67 years, respectively)^19^, suggesting that the present study included more children and adolescents with primary NS as compared with JNSCS. In contrast, the male predominance for all primary NS subtypes in the present study is similar to the previous findings^3,6,19,20^. In Japan, JNSCS reported that 41.4% had MCD, 10.2% had FSGS, 24.4% had MN, and 8.8% had other types. In the J-RBR, MCD, FSGS, and MN comprised 40.0–45.7%, 11.3–13.0%, and 35.6% of the total number of kidney biopsies^3,20^. Compared with these previous studies in Japan, the present study found that MCD (56.2%) was more dominant, whereas MN (24.1%) was less, suggesting that a younger age of onset in MCD than those in previous studies may be due to etiologic differences. Compared to Nephrotic Syndrome Study Network (NEPTUNE) in North America (27% MCD, 32% FSGS, 15% MN, and 27% other glomerulopathies)^4^, the present study shows that the prevalence of FSGS in Japan was lower (11.4%) and has not changed in the past decade^21^. These epidemiological differences could be attributed partially to the genetic variants in *apolipoprotein L1 (APOL1)* among people with sub-Saharan ancestry^22^.

In Analysis 2–1, we compared the demographics, clinical features, and treatment of the three major pathological types of primary NS, which are MCD, FSGS, and MN. To the best of our knowledge, this is the first report to describe both the use and the mean dosage of oral prednisolone was significantly higher in MCD (87.2%, 21.2 ± 17.8 mg/day) than in MN (77.5%, 18.8 ± 15.4 mg/day), possibly because SDNS or FRNS was significantly higher in MCD than in MN. In addition, since infectious disease is a harmful complication among elderly NS patients, the MN patients were older than the MCD patients and may have received a reduced dose of steroids to prevent infection. These findings are consistent with JNSCS demonstrating better remission rates in elderly patients with NS^9^. Accordingly, future clinical guideline for elderly patients with NS should be developed to reflect new findings.

The median age of onset and the median age at renal biopsy were lower for MCD than for FSGS and MN (31 vs. 39 and 60 years, respectively; 34 vs. 41 and 61 years, respectively). In adult patients with NS, renal biopsy is mandatory for diagnosis if the cause is not apparent on initial evaluation^2^. However, in children with steroid-sensitive NS, the clinical presentation is usually sufficiently characteristic to guide initial management without biopsy^23^. Therefore, the difference between the age of onset and the age at renal biopsy might be greater in patients with MCD than in those with FSGS and MN, especially in younger patients with all three glomerulopathies.

Compared with the JNSCS^6^, the rates of prescription of oral prednisolone (98.7% vs. 87.2%) and intravenous methylprednisolone (31.6% vs. 7.7%) were lower, whereas the rates of prescription of cyclosporine (31.6% vs. 47.2%), mizoribine (5.3% vs. 12.2%), rituximab (2.6% vs. 6.0%), and mycophenolate mofetil (0.7% vs. 2.6%) were higher in patients with MCD in the present study, suggesting that the patients with clinically more acute and severe cases were preferentially included in the JNSCS. Furthermore, the present study might include many patients with remission using multiple immunosuppressive drugs.

Compared with MCD, the prescription rates of intravenous methylprednisolone, cyclosporine, tacrolimus, and mycophenolate mofetil were higher in FSGS, as the incidence of steroid-resistant NS was significantly higher in FSGS than in MCD (36.0% and 6.4%, respectively). Compared with J-RBR^11^, the prescription rates of immunosuppressive drugs were higher in patients with FSGS in the present study (oral prednisolone [53% vs. 80.9%], cyclosporine [33% vs. 51.5%], tacrolimus [2% vs. 4.0%], mycophenolate mofetil [2% vs. 4.4%], rituximab [1% vs. 5.8%]), suggesting that occurrence of steroid-resistant FSGS was higher in the present study than in J-RBR.

Analysis 2–2 in this study included the largest number of elderly (65–74 years) or very elderly (> 75 years) onset patients, i.e. 212 and 118 patients with MCD, 61 and 25 with FSGS, and 363 and 173 with MN, respectively. Elderly and very elderly onset patients with MCD and FSGS have a higher prescription rate and higher dose of steroids and immunosuppressive drugs than younger patients, suggesting that steroid-resistant NS was much common even in the elderly or very elderly patients with MCD and FSGS. In contrast, patients with MN were observed to have similar medications and similar percentages of steroid-resistant NS among the five age groups.

As for child and adolescent groups of patients with MCD and FSGS, the use of oral prednisolone and intravenous methylprednisolone was lower than that in the other age groups, which might in turn be associated with a higher use of rituximab for SDNS and FRNS in pediatric and adolescent patients with MCD and FSGS as compared with the other age groups. Rituximab has been demonstrated to be effective and safe in patients with difficult-to-treat SDNS and/or FRNS, for reducing the relapse frequency and maintaining remission despite the cessation or tapering of steroids^24^. This study has attemped to revel the real-world use of rituximab for trating primary NS throughout Japan.

The Japanese clinical guideline for NS, published in 2014, recommends that patients with relapsing patients of MCD or steroid-resistant NS should be administered the combination treatment of cyclosporine and steroid as effective for reducing urinary protein levels and shortening the duration of achieving remission, compared to steroid-alone treatment (Recommendation grade: C1)^25^. The present study has described the QIs related to the primary NS treatment, showing that the use of prednisolone + cyclosporine combination therapy in steroid‑resistant MCD, FSGS, and MN by 42.7%, 47.4%, and 42.2%, respectively, as present treatment, and 51.6%, 67.8%, and 58.6%, respectively, as previous treatment. Moreover, use of the same combination therapy in steroid-dependent or frequently relapsing MCD accountd for 48.8% in the present treatments and 71.0% in the previous treatments. The evaluation of QIs related to the treatment of primary NS is believed to improve the outcome.

This study has several limitations. First, the database does not include clinical characteristics observed at renal biopsy, such as urine protein levels, estimated glomerular filtration rate, serum albumin, and detailed pathological findings including immunostaining. Furthermore, the database does not contain data about complications (hypertension and diabetes), genetic testing, nonpharmacological therapy, cumulative dose of medication, duration from initial renal biopsy, and patient outcomes. We could obtain the renal biopsy date but could not determine whether it was an initial or a repeat biopsy. The data were collected from personal clinical records, and their accuracy depended mainly on those who completed the forms; however, we assessed the validity of the data to the best of our ability. The validity of the analysis itself should be evaluated in further studies using data from other cohorts. Second, we demonstrated the characteristics of primary NS throughout Japan using descriptive analysis. However, a temporal trend analysis would provide more clinically relevant findings. Further studies using temporal trend analysis are needed to reveal longitudinal demographic changes and the rate of guideline-directed medical treatments. Despite these limitations, we believe that the outcomes of this study will help provide an insightful overview of the clinical characteristics and features of patients with primary NS throughout Japan, in addition to developing the optimal management for these patients.

In conclusion, by analyzing a nationwide database of more than 6000 patients with primary NS, this study revealed the clinical characteristics of MCD, FSGS, and MN in Japan and provided critical insights for developing optimal management strategies for these glomerulopathies. The gap between current clinical practice and guideline recommendations should be discussed, and further investigations are needed to improve the therapeutic strategies against primary NS in Japan.

## Methods

### Overviews of clinical personal records

The National Database of Designated Incurable Diseases of Japan was established in Japan and consists of data from the clinical personal records voluntarily submitted when individuals apply for medical support^26^. These clinical personal records contain clinically significant information such as symptoms, laboratory test results, and histopathological findings^27^. Since the Act on Medical Care for Patients with Intractable Diseases was enforced in 2015, data on 338 diseases (as of September 2022), including primary NS (designated intractable disease number 222), IgA nephropathy (designated intractable disease number 66), rapidly progressive glomerulonephritis (designated intractable disease number 220), and polycystic kidney disease (designated intractable disease number 67) collected from clinical personal records have been added to the database.

This cross-sectional study used data from clinical personal records of primary NS, throughout Japan, maintained by the Japanese Ministry of Health, Labour, and Welfare. The records prospectively and annually collected demographic data (age, sex), histological diagnosis (MCD, FSGS, FSGS, membranoproliferative glomerulonephritis, crescentic glomerulonephritis, endocapillary proliferative glomerulonephritis, unknown or unclassifiable), chronic kidney disease classification based on glomerular filtration rate and albuminuria, date of renal biopsy, medication use (presently, previously used, and maximum dosage of present treatment within 6 months), and therapeutic responses (steroid-resistant [never achieved remission], dependent or frequently relapsing). The data were registered only after being reviewed by certified nephrologists^27^. However, the database does not include survival data, such as death. The results presented in this study are original and different from the statistics produced or published by the Ministry of Health, Labour and Welfare of Japan^27^.

### Study population

The present study used clinical personal records of patients with primary NS from 2015 to 2018. Primary NS was diagnosed based on massive proteinuria (≥ 3.5 g/day) and hypoalbuminemia (serum albumin ≤ 3.0 g/dL) in the absence of any secondary nephrotic syndrome^1^, such as autoimmune diseases (lupus nephritis, IgA vasculitis, and other types of vasculitis), diabetic nephropathy, paraproteinemia (amyloidosis, cryoglobulin, heavy chain deposition, and light chain deposition), infectious diseases (streptococcal and staphylococcal infections, hepatitis B and C, human immunodeficiency virus, parvovirus B19, syphilis, parasites [malaria and schistosomiasis]), tumors (solid tumors, multiple myeloma, malignant lymphoma, and leukemia), drugs (bucillamine, D-penicillamine, and gold), genetic diseases (Alport syndrome, Fabry disease, and nail-patella syndrome), pregnancy hypertension nephropathy, radiation nephropathy, or rejection of the transplanted kidney.

### Data curation

Data used in stasitical analyses had to be processed and refined in advance to render them suitable for analysis^26,27^. If multiple patients had identical data, the data of one patient was included for analysis and the duplicate data was excluded. Erroneous data (e.g. values that were entered although no tests were performed) were included after likely values were assigned as alternatives. These likely values were determined by referring to the basic statistics of the data^26,27^.

### Characteristics and management of primary NS

The clinical characteristics and management of each primary NS in this registry were determined. The use and dosage (highest dose in the past 6 months; present use only) of present and previously used steroids and immunosuppressive drugs were also collected. The response of NS to treatment was assessed according to the definition outlined by the Japanese guidelines for NS^25^ as follows. Steroid-resistant NS is defined as never achieved complete remission despite immunosuppressive treatment, including steroid therapy, after diagnosis of NS. SDNS is defined as when steroids cannot be discontinued because of two or more relapses after reducing or discontinuing the dose of steroids. FRNS is defined by frequent relapses (2 or more relapses in 6 months).

### Analysis set

Analysis 1: Distribution and frequency of glomerulopathies in the national registry of clinical personal records of primary NS by age (in years) of onset.

Analysis 2–1: Comparison of demographics, clinical features, and treatment modalities between MCD, FSGS, and MN after removing patients with multiple diagnoses and missing data (Fig. 1).

Analysis 2–2: Patients with MCD, FSGS, and MN were divided into five age groups at onset: child and adolescent (≤ 19 years), young adult group (20–39 years), middle adult group (40–64 years), elderly group (65–74 years), and very elderly group (≥ 75 years). Clinical features were compared among the age groups.

### QIs related to the treatment of primary NS

We investigated the QIs related to the treatment for primary NS in some clinical questions (CQs) in Japanese guidelines for nephrotic syndrome “Evidence-based clinical practice guidelines for nephrotic syndrome 2014”^25^.

- *CQ5.* Is the addition of immunosuppressive agents to steroids recommended for reducing urinary protein levels or preventing renal function decline in frequently relapsing MCD?
- *CQ6.* Are additional immunosuppressive agents to steroids recommended for reducing the urinary protein level and preventing renal function decline in steroid-resistant FSGS?
- *CQ9.* Is cyclosporine recommended to reduce the urinary protein level and prevent renal function decline in MN?

### Statistical analysis

Quantitative variables have been expressed as mean (standard deviation) for normally distributed data or median (IQR). Qualitative variables are presented as frequencies (percentages). Differences between groups were compared using chi-squared tests for categorical variables and nonparametric Kruskal–Wallis tests for continuous variables. All data were analyzed using IBM SPSS (version 26.0; SPSS, Chicago, IL, USA), and P < 0.05 indicated a significant difference.

### Ethics statement

The study protocol was approved by the Ethics Committee of the Japanese Society of Nephrology (approval number 70), the Institutional Review Board of Niigata University, Asahikawa Medical University, and National Institutes of Biomedical Innovation, Health and Nutrition. Written informed consent to register the data of clinical personal records in the database and to use the data for research and policy-making was obtained from all patients. All procedures performed in the present study were in accordance with the Declaration of Helsinki.

### Supplementary Information

Supplementary Information.

## Data Availability

The data that support the findings of this study are available from the working group on data provision of the Ministry of Health, Labour, and Welfare, Japan; but access to the data, which was used under license for the current study is restricted, and so is not publicly available. However, data can be obtained from the corresponding author upon reasonable request, and with the permission of the working group on data provision of the Ministry of Health, Labour, and Welfare, Japan.
