# Supplementary material for: Demographics and treatment of patients with primary nephrotic syndrome in Japan using a national registry of clinical personal records
Source: Sci Rep. 2023 Sep 7;13:14771. doi: 10.1038/s41598-023-41909-5 (PMC10485053; doi:10.1038/s41598-023-41909-5)
Supplement: Supplementary file 1 — Supplementary Information. [file 41598_2023_41909_MOESM1_ESM.docx]

**Demographics and Treatment of Patients with Primary Nephrotic Syndrome in Japan using a National Registry of Clinical Personal Records**

Naoki Nakagawa^1^*, Tomonori Kimura^2,3^, Ryuichi Sakate^2^, Takehiko Wada^4,5^, Kengo Furuichi^6^, Hirokazu Okada^7^, Yoshitaka Isaka^8^, and Ichiei Narita^9^

^1^Division of Cardiology, Nephrology, Pulmonology and Neurology, Department of Internal Medicine, Asahikawa Medical University, Asahikawa, Japan

^2^Reverse Translational Research Project, Center for Rare Disease Research, National Institutes of Biomedical Innovation, Health and Nutrition (NIBIOHN), Ibaraki, Japan

^3^Laboratory of Rare Disease Resource Library, Center for Rare Disease Research, National Institutes of Biomedical Innovation, Health and Nutrition (NIBIOHN), Ibaraki, Japan

^4^Division of Nephrology, Endocrinology and Metabolism, Tokai University School of Medicine, Isehara, Japan

^5^Department of Nephrology, , Toranomon Hospital, Tokyo, Japan

^6^Department of Nephrology, Kanazawa Medical University School of Medicine, Ishikawa, Japan

^7^Department of Nephrology, Faculty of Medicine, Saitama Medical University, Saitama, Japan

^8^Department of Nephrology, Osaka University Graduate School of Medicine, Suita, Japan

^9^Division of Clinical Nephrology and Rheumatology, Kidney Research Center, Niigata University Graduate School of Medical and Dental Sciences, Niigata, Japan

*Corresponding author: Naoki Nakagawa, MD, PhD, Division of Cardiology, Nephrology, Pulmonology and Neurology, Department of Internal Medicine, Asahikawa Medical University, 2-1-1-1 Midorigaoka-higashi, Asahikawa, Japan, Phone: +81-166-68-2442, Fax: +81-166-68-2449, E-mail: naka-nao@asahikawa-med.ac.jp (NN)

Supplementary Figure S1. Risk classification of chronic kidney disease by age of onset in patients with MCD, FSGS, and MN


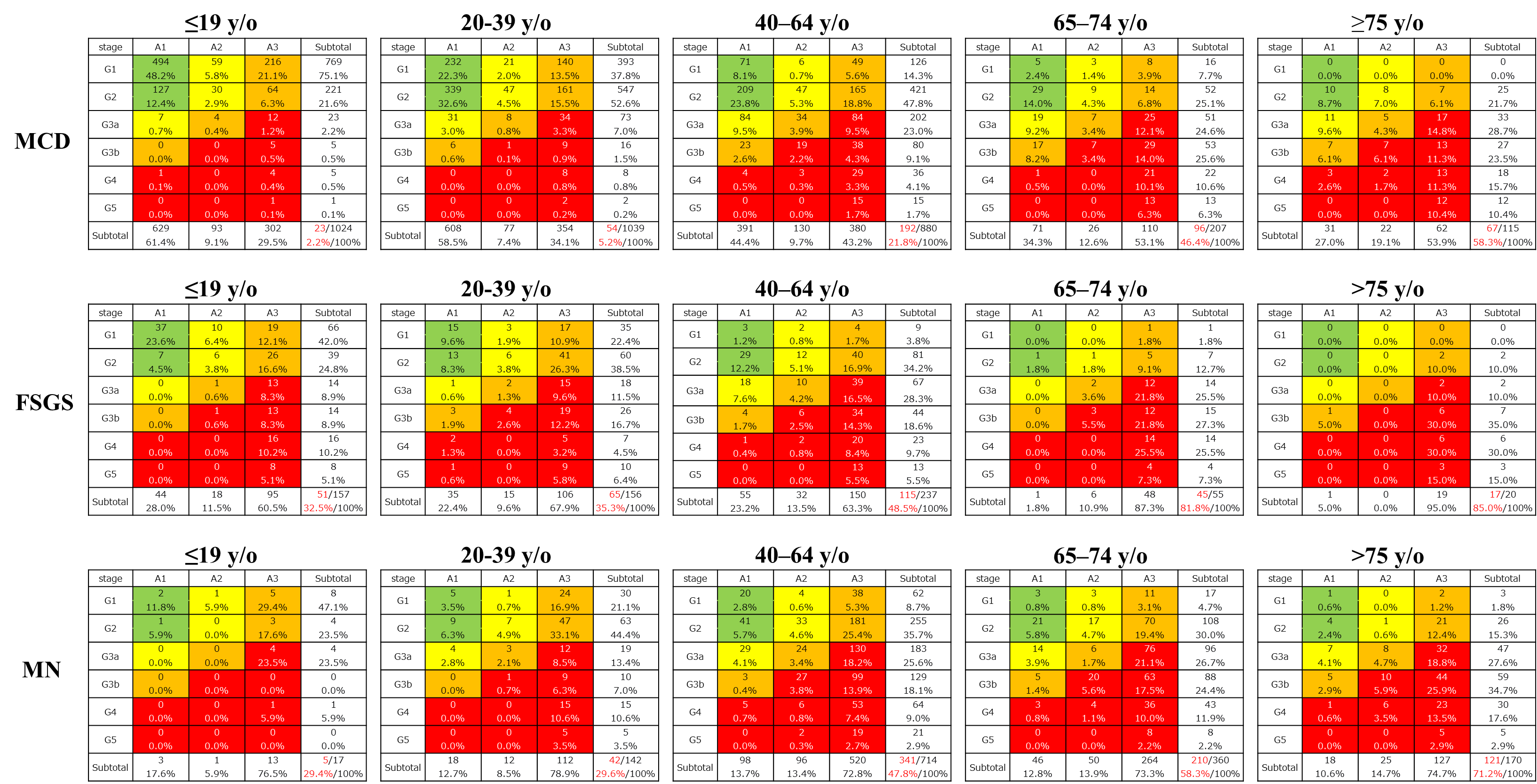


MCD, minimal change disease; FSGS, focal segmental glomerulosclerosis; MN, membranous nephropathy.

Supplementary Table S1. Demographics of patients with steroid-resistant nephrotic syndrome.

|  | MCD | FSGS | MN | P value |
| --- | --- | --- | --- | --- |
| N | 213 | 236 | 540 |  |
| Age of onset (years old) | 51 (34, 67) | 43 (25, 63) | 60 (48, 69) | <0.001 ^a,b,c^ |
| Age at renal biopsy (yrs)* | 53 (39, 67) | 47 (32, 64) | 62 (50, 70) | <0.001 ^a,b,c^ |
| Age of enrollment (years old) | 53 (41, 68) | 49 (36, 65) | 66 (57, 73) | <0.001 ^b,c^ |
| Male, n (%) | 125 (58.7%) | 137 (58.1%) | 358 (66.3%) | 0.036 ^c^ |
| CKD G stage |  |  |  |  |
| G1 | 44 (20.7%) | 27 (11.4%) | 40 (7.4%) | <0.001 ^a,b^ |
| G2 | 67 (31.5%) | 56 (23.7%) | 162 (30.0%) |  |
| G3a | 32 (15.0%) | 43 (18.2%) | 129 (23.9%) |  |
| G3b | 25 (11.7%) | 46 (19.5%) | 116 (21.5%) |  |
| G4 | 23 (10.8%) | 44 (18.6%) | 68 (12.6%) |  |
| G5 | 22 (10.3%) | 20 (8.5%) | 25 (4.6%) |  |
| CKD A stage |  |  |  |  |
| A1 | 21 (9.9%) | 4 (1.7%) | 10 (1.9%) | <0.001 ^a,b^ |
| A2 | 12 (5.6%) | 7 (3.0%) | 34 (6.3%) |  |
| A3 | 180 (84.5%) | 225 (95.3%) | 496 (91.9%) |  |
| Present treatment |  |  |  |  |
| Oral prednisolone | 186 (87.3%) | 205 (86.9%) | 448 (83.0%) | 0.197 |
| IV methylprednisolone | 46 (21.6%) | 34 (14.4%) | 25 (4.6%) | <0.001 ^a,b,c^ |
| Cyclosporine | 97 (45.5%) | 124 (52.5%) | 271 (50.2%) | 0.319 |
| Tacrolimus | 2 (0.9%) | 13 (5.5%) | 9 (1.7%) | 0.001 ^a,c^ |
| Cyclophosphamide | 2 (0.9%) | 2 (0.8%) | 15 (2.8%) | 0.099 |
| Mizoribine | 23 (10.8%) | 40 (16.9%) | 123 (22.8%) | 0.001 ^b^ |
| Mycophenolate mofetil | 2 (0.9%) | 9 (3.8%) | 0 (0.0%) | <0.001 ^a,c^ |
| Rituximab | 10 (4.7%) | 16 (6.8%) | 5 (0.9%) | <0.001 ^b,c^ |
| Prednisolone + cyclosporine | 91 (42.7%) | 112 (47.5%) | 237 (43.9%) | 0.454 |
| Dosage of present treatment |  |  |  |  |
| Oral prednisolone (mg/day) | 32.1 ± 18.1 | 21.8 ± 17.3 | 22.1 ± 15.9 | <0.001 ^a,b^ |
| IV methylprednisolone (mg/day) | 598.0 ± 292.3 | 545.5 ± 146.0 | 571.9 ± 249.1 | 0.651 |
| Cyclosporine (mg/day) | 97.5 ± 34.6 | 100.4 ± 42.0 | 98.2 ± 37.2 | 0.997 |
| Tacrolimus (mg/day) | 3.5 ± 2.1 | 3.9 ± 2.6 | 2.1 ± 0.5 | 0.122 |
| Cyclophosphamide (mg/day) | 87.5 ± 17.7 | 100.0 ± 70.7 | 70.0 ± 23.5 | 0.512 |
| Mizoribine (mg/day) | 167.5 ± 65.4 | 152.3 ± 73.8 | 143.2 ± 47.0 | 0.135 |
| Mycophenolate mofetil (mg/day) | 875.0 ± 883.9 | 1389.0 ± 771.5 | - | 0.620 |
| Rituximab (mg/month) | 500.0 ± 0.0 | 697.3 ± 529.0 | 500.0 ± 0.0 | 0.866 |
| Previous treatment |  |  |  |  |
| Oral prednisolone | 182 (85.4%) | 213 (90.3%) | 458 (85.0%) | 0.131 |
| IV methylprednisolone | 80 (37.6%) | 123 (52.1%) | 102 (18.9%) | <0.001 ^a,b,c^ |
| Cyclosporine | 111 (52.1%) | 166 (70.3%) | 338 (62.6%) | <0.001 ^a,b^ |
| Tacrolimus | 2 (0.9%) | 19 (8.1%) | 8 (1.5%) | <0.001 ^a,c^ |
| Cyclophosphamide | 15 (7.0%) | 15 (6.4%) | 31 (5.7%) | 0.792 |
| Mizoribine | 35 (16.4%) | 59 (25.0%) | 164 (30.4%) | <0.001 ^b^ |
| Mycophenolate mofetil | 4 (1.9%) | 11 (4.7%) | 2 (0.4%) | <0.001 ^c^ |
| Rituximab | 10 (4.7%) | 23 (9.7%) | 4 (0.7%) | <0.001 ^a,b,c^ |
| Prednisolone + cyclosporine | 110 (51.6%) | 160 (67.8%) | 316 (58.6%) | 0.001 ^a,c^ |
| Persistent proteinuria ≥0.5 g/gCr | 119 (55.6%) | 186 (78.8%) | 367 (68.0%) | <0.001 ^a,b,c^ |

Data are expressed as mean ± SD, median (interquartile range) or number (percentage). ^a^P < 0.05, MCD vs. FSGS. ^b^P < 0.05, MCD vs. MN. ^c^P < 0.05, FSGS vs. MN. Kruskal–Wallis tests with Bonferroni-corrected P-values.

*Number of missing values, n = 15 (7.0%) in MCD; n = 10 (4.2%) in FSGS; n = 16 (3.0%) in MN.

MCD, minimal change disease; FSGS, focal segmental glomerulosclerosis; MN, membranous nephropathy.

Supplementary Table S2. Demographics of patients with SDNS or FRNS

|  | MCD | FSGS | MN | P value |
| --- | --- | --- | --- | --- |
| N | 2320 | 265 | 351 |  |
| Age of onset (years old) | 25 (14, 40) | 34 (14, 49) | 56 (45, 64) | <0.001 ^a,b,c^ |
| Age at renal biopsy (yrs)* | 29 (18, 43) | 38 (21, 52) | 57 (49, 65) | <0.001 ^a,b,c^ |
| Age of enrollment (years old) | 39 (27, 51) | 44 (31, 59) | 67 (58, 73) | <0.001 ^a,b,c^ |
| Male, n (%) | 1349 (58.1%) | 158 (59.6%) | 221 (63.0%) | 0.224 |
| CKD G stage |  |  |  |  |
| G1 | 1041 (44.9%) | 69 (26.0%) | 25 (7.1%) | <0.001 ^a,b,c^ |
| G2 | 946 (40.8%) | 93 (35.1%) | 135 (38.5%) |  |
| G3a | 234 (10.1%) | 46 (17.4%) | 94 (26.8%) |  |
| G3b | 73 (3.1%) | 37 (14.0%) | 55 (15.7%) |  |
| G4 | 20 (0.9%) | 14 (5.3%) | 42 (12.0%) |  |
| G5 | 6 (0.23%) | 6 (2.23%) | 0 (0.0%) |  |
| CKD A stage |  |  |  |  |
| A1 | 1386 (59.7%) | 104 (39.2%) | 77 (21.9%) | <0.001 ^a,b,c^ |
| A2 | 252 (110.9%) | 40 (15.1%) | 73 (20.8%) |  |
| A3 | 682 (29.4%) | 121 (45.7%) | 201 (57.3%) |  |
| Present treatment |  |  |  |  |
| Oral prednisolone | 2075 (89.4%) | 232 (87.5%) | 310 (88.3%) | 0.562 |
| IV methylprednisolone | 124 (5.3%) | 28 (10.6%) | 6 (1.7%) | <0.001 ^a,b,c^ |
| Cyclosporine | 1247 (53.8%) | 162 (61.1%) | 172 (49.0%) | 0.011 ^c^ |
| Tacrolimus | 47 (2.0%) | 10 (3.8%) | 3 (0.9%) | 0.040 ^c^ |
| Cyclophosphamide | 10 (0.4%) | 1 (0.4%) | 3 (0.9%) | 0.545 |
| Mizoribine | 353 (15.2%) | 39 (14.7%) | 72 (20.5%) | 0.035 ^b^ |
| Mycophenolate mofetil | 79 (3.4%) | 18 (6.8%) | 0 (0.0%) | <0.001 ^a,b,c^ |
| Rituximab | 184 (7.9%) | 26 (9.8%) | 4 (1.1%) | <0.001 ^b,c^ |
| Prednisolone + cyclosporine | 1132 (48.8%) | 145 (47.4%) | 148 (42.2%) | 0.024 ^c^ |
| Dosage of present treatment |  |  |  |  |
| Oral prednisolone (mg/day) | 17.6 ± 15.5 | 16.6 ± 14.1 | 11.6 ± 11.0 | 0.001 ^b,c^ |
| IV methylprednisolone(mg/day) | 495.5 ± 330.5 | 518.8 ± 258.4 | 282.5 ± 255.9 | 0.184 |
| Cyclosporine (mg/day) | 96.5 ± 40.6 | 93.8 ± 41.5 | 81.6 ± 33.9 | <0.001 ^b,c^ |
| Tacrolimus (mg/day) | 2.3 ± 0.8 | 3.5 ± 2.3 | 3.0 ± 0.0 | 0.031 |
| Cyclophosphamide (mg/day) | 67.5 ± 39.2 | 50.0 | 50.0 ± 0.0 | 0.661 |
| Mizoribine (mg/day) | 151.4 ± 68.1 | 143.8 ± 55.5 | 135.5 ± 42.9 | 0.259 |
| Mycophenolate mofetil (mg/day) | 1458.9 ± 513.3 | 1583.4 ± 624.1 | - | 0.362 |
| Rituximab (mg/month) | 597.4 ± 405.8 | 845.2 ± 610.9 | 500.0 ± 0.0 | 0.028 ^a^ |
| Previous treatment |  |  |  |  |
| Oral prednisolone | 2188 (94.3%) | 245 (92.5%) | 318 (90.6%) | 0.019 ^b^ |
| IV methylprednisolone | 927 (40.0%) | 152 (57.4%) | 67 (19.1%) | <0.001 ^a,b,c^ |
| Cyclosporine | 1700 (73.3%) | 218 (82.3%) | 225 (64.1%) | <0.001 ^a,b,c^ |
| Tacrolimus | 80 (3.4%) | 18 (6.8%) | 6 (1.7%) | 0.003 ^a,c^ |
| Cyclophosphamide | 216 (9.3%) | 31 (11.7%) | 23 (6.6%) | 0.084 |
| Mizoribine | 688 (29.7%) | 84 (31.7%) | 104 (29.6%) | 0.786 |
| Mycophenolate mofetil | 112 (4.8%) | 22 (8.3%) | 0 (0.0%) | <0.001 ^a,b,c^ |
| Rituximab | 206 (8.9%) | 31 (11.7%) | 2 (0.6%) | <0.001 ^b,c^ |
| Prednisolone + cyclosporine | 1647 (71.0%) | 211 (79.6%) | 210 (59.8%) | <0.001 ^a,b,c^ |
| Persistent proteinuria ≥0.5 g/gCr | 198 (8.5%) | 69 (26.0%) | 140 (39.9%) | <0.001 ^a,b,c^ |

Data are expressed as mean ± SD, median (interquartile range) or number (percentage). ^a^P < 0.05, MCD vs. FSGS. ^b^P < 0.05, MCD vs. MN. ^c^P < 0.05, FSGS vs. MN. Kruskal–Wallis tests with Bonferroni-corrected P-values.

*Number of missing values, n = 252 (10.9%) in MCD; n = 13 (4.9%) in FSGS; n = 12 (3.4%) in MN.

MCD, minimal change disease; FSGS, focal segmental glomerulosclerosis; MN, membranous nephropathy; SDNS, steroid-dependent nephrotic syndrome; FRNS, frequently relapsing nephrotic syndrome.

Supplementary Table 3. Quality indicators related to the treatment for primary NS

|  | Steroid-resistant NS | | | SDNS or FRNS |
| --- | --- | --- | --- | --- |
|  | MCD | FSGS | MN | MCD |
| N | 213 | 236 | 540 | 2320 |
| Present treatment |  |  |  |  |
| Prednisolone + cyclosporine, n (%) | 91 (42.7%) | 112 (47.4%) | 237 (42.2%) | 1132 (48.8%) |
| Previous treatment |  |  |  |  |
| Prednisolone + cyclosporine, n (%) | 110 (51.6%) | 160 (67.8%) | 316 (58.6%) | 1647 (71.0%) |

Data are expressed as number (percentage). MCD, minimal change disease; FSGS, focal segmental glomerulosclerosis; MN, membranous nephropathy; SDNS, steroid-dependent nephrotic syndrome; FRNS, frequently relapsing nephrotic syndrome.
